# A Metabolomics-Based Strategy for Identifying Endogenous Inhibitors of IAPP Aggregation

**DOI:** 10.3390/metabo16080549

**Published:** 2026-08-03

**Authors:** Dandan Xia, Liubao Gu, Lei Yang, Jiaojiao Hu, Xiaowei Xu, Dechen Jiang, Qiuling Zheng, Bing Wan

**Affiliations:** 1 Department of Respiratory and Critical Care Medicine, The Affiliated Jiangning Hospital of Nanjing Medical University, Nanjing 211100, China; dg21240118@smail.nju.edu.cn; 2 The State Key Lab of Analytical Chemistry for Life Science, Chemistry and Biomedicine Innovation Center (ChemBIC), School of Chemistry and Chemical Engineering, Nanjing University, Nanjing 210093, China; 3 Department of Pharmaceutical Analysis, School of Pharmacy, China Pharmaceutical University, Tongjiaxiang #24, Nanjing 210009, China; 4 Division of Clinical Epidemiology, Geriatric Hospital of Nanjing Medical University, Nanjing 210009, China; liubao_gu@njmu.edu.cn; 5 Institute of Innovative Drug Discovery and Development, China Pharmaceutical University, Tongjiaxiang #24, Nanjing 210009, China

**Keywords:** metabolomic analysis, IAPP aggregation, mass spectrometry

## Abstract

**Background/Objectives**: Human islet amyloid polypeptide (IAPP) aggregation plays a critical role in the pathogenesis of type 2 diabetes mellitus (T2DM). Although metabolic alterations are a hallmark of T2DM, the functional roles of differential metabolites in regulating disease-associated molecular processes remain largely unexplored. This study aimed to establish a metabolomics-guided strategy for identifying endogenous metabolites with anti-amyloid activity and to investigate their underlying chemical interactions with IAPP. **Methods**: Untargeted metabolomic profiling of clinical samples from T2DM patients, obesity patients and healthy controls was performed to identify differential metabolites. Candidate metabolites were subsequently screened for their ability to modulate IAPP aggregation. Transmission electron microscopy (TEM), thioflavin T (ThT) fluorescence assays, and cell viability measurements were employed to evaluate their effects on fibril formation and cytotoxicity. Mass spectrometry was further used to characterize metabolite–IAPP interactions. **Results**: Metabolomic analysis identified 3-hydroxypyruvic acid (also known as β-hydroxypyruvic acid, hereafter referred to as HPA) as a significantly altered endogenous metabolite associated with T2DM and a candidate regulator of IAPP aggregation. Functional assays demonstrated that HPA effectively inhibited amyloid fibril formation, as evidenced by the absence of typical fibrillar structures and a prolonged lag phase during aggregation. HPA also significantly alleviated IAPP-induced cytotoxicity. Mass spectrometric analysis revealed the formation of HPA–IAPP oligomer complexes (*n* < 4), suggesting that HPA directly interacts with early oligomeric intermediates and interferes with their progression toward mature fibrils. **Conclusions**: This work demonstrates that untargeted metabolomics of clinical samples can serve as an effective strategy for discovering bioactive endogenous metabolites involved in disease-related molecular processes. The identification of HPA as a potential endogenous inhibitor of IAPP aggregation provides new chemical insight into the relationship between metabolic dysregulation and amyloidogenesis and highlights endogenous metabolites as a valuable source of potential therapeutic lead compounds.

## 1. Introduction

IAPP is a polypeptide hormone consisting of 37 amino acids [1], which has the physiological functions of slowing gastric emptying and reducing appetite, as well as cooperating with insulin and glucagon to accurately regulate blood glucose levels [2]. Under certain pathological conditions, IAPP monomers can misfold and aggregate, resulting in the formation of amyloid deposits in pancreatic islets, which have been identified in the majority of pancreatic islet tissue samples from patients with T2DM. The formation of toxic IAPP aggregates is likely one of the contributing factors to β-cell failure [3,4]. Metabolic disorders in T2DM, such as abnormal fluctuations in glucose and lipid levels, are associated with increased IAPP secretion [5,6]. Recent studies also report that early-stage oligomers, rather than mature fibrils, are the primary contributors to cytotoxicity and β-cell dysfunction [7,8]. Therefore, pathologically induced IAPP aggregation and fibrillation are closely related to the underlying pathology of T2DM [9]. The inhibition of amyloid aggregation of IAPP is essential for the prevention and treatment of T2DM.

Great efforts have been made to explore effective treatments for amyloid-related diseases aimed at delaying, inhibiting or even reversing fibril formation, including nanoparticles and immunotherapeutic targeting of amyloid deposits and amyloid protein mimics [10,11,12,13,14,15]. In addition, small-molecule inhibitors have attracted great attention, and numerous studies have demonstrated their ability in vitro to inhibit fibril formation and reduce cytotoxicity [16]. For instance, epigallocatechin-3-gallate (EGCG), a flavonoid extracted from green tea, has been shown to alter the aggregation pathway and lead to the formation of nontoxic amorphous aggregates [17,18,19]. Nevertheless, the poor intestinal absorption and instability impede its applications [20]. Currently available small-molecule inhibitors are either chemically synthesized or extracted from natural products. However, their in vivo efficacy remains limited due to their relatively low bioavailability. Therefore, the exploration of novel and effective inhibitors for IAPP aggregation is urgently required.

Over the past two decades, metabolomics has become an important tool for disease biomarker discovery and pathway analysis, contributing significantly mechanistic studies of disease pathogenesis [21,22,23]. In this study, we proposed that there may be endogenous metabolites with potential inhibitory effects on IAPP aggregation that remain to be discovered. Accordingly, untargeted metabolomic analysis was first employed to discover differential metabolites, focusing on those associated with T2DM, and the effects of these metabolites on the IAPP aggregation process were monitored. On this basis, HPA, which was previously reported as a diabetic differential metabolite [24,25], emerged as a potential inhibitor. This was observed through the absence of typical fibril formation, as characterized by TEM morphological analysis. A ThT fluorescence assay was then employed to monitor aggregation kinetics, and the results indicated that HPA effectively delayed the lag phase of IAPP aggregation, contrasting with the aggregation of IAPP in the absence of HPA. Moreover, the reduction in IAPP-induced cytotoxicity upon the addition of HPA provided additional evidence that oligomers could be a possible target of HPA. Furthermore, cellular immunofluorescence studies revealed that HPA mitigated the IAPP-induced apoptosis by reducing the expression of cleaved caspase-3 while preserving nuclear integrity. On this basis, we proposed that HPA exerts its inhibitory effect by interacting with monomeric or oligomeric forms of IAPP. Mass spectrometry (MS) investigations revealed that HPA interacted with IAPP oligomers of lower oligomeric states (*n* < 4) and prevented their progression to higher order states, which could be the underlying mechanism [26]. Therefore, the present study suggests that HPA has the potential to delay IAPP aggregation and may serve as a promising drug precursor. These findings offer new insights into the discovery of inhibitors derived from differential endogenous metabolites and contribute to the design and development of new inhibitors.

## 2. Materials and Methods

### 2.1. Chemicals and Reagents

HPA, ThT, 1,1,1,3,3,3-Hexafluoro-2-propanol (HFIP), and 3-hydroxybutyrate were all purchased from Sigma Aldrich (St. Louis, MO, USA). 3-N-Methyl-L-histidine and thiazolyl blue tetrazolium bromide were purchased from Aladdin (Los Angeles, CA, USA). 1640 medium, fetal bovine serum, donkey anti-rabbit IgG (H + L) highly cross-adsorbed secondary antibody, and Alexa Fluor Plus 555 were purchased from Thermo Fisher Scientific (Waltham, MA, USA). RIN-m5F cell was purchased from Jennio Biotech (Guangzhou, China). IAPP was purchased from Nanjing Peptide Industry (Nanjing, China). Cleaved caspase-3 antibody was purchased from Cell Signaling Technology (Danvers, MA, USA). Hoechst 33342, 4% Paraformaldehyde fix solution, Triton X-100, Poly-D-lysine, and bovine serum albumin were purchased from Beyotime (Shanghai, China). Acetonitrile was purchased from Merck (Darmstadt, Germany), and deionized water was prepared using a Milli-Q system of Millipore (Billerica, MA, USA).

### 2.2. Metabolomic Profiling

Serum samples were obtained from 9 healthy people, 8 T2DM patients and 10 people with obesity for metabolomic analysis (Table S1). The study was conducted in accordance with the Declaration of Helsinki and was approved by the Ethical Committee of the Affiliated Geriatric Hospital of Nanjing Medical University (Project identification code: M2020099; Ethics approval No. (2020) 020) on 27 August 2020. Written informed consent was obtained from all participants. Metabolomic analysis was performed by using a Shimadzu CBM30A Ultra Performance Liquid Chromatography system (Kyoto, Japan) equipped with a Waters Acquity UPLC HSS C18 column (2.1 mm × 100 mm, 1.8 μm) (Milford, MA, USA). The LC eluent was analyzed using a QTRAP 6500+ mass spectrometer manufactured by AB SCIEX (Framingham, MA, USA). Qualitative analysis was performed based on the retention time, ion pair information, and the MS/MS spectrum data of the detected substance using a locally developed MetWare database (MWDB). According to the Metabolomics Standards Initiative (MSI) guidelines, metabolite identifications were assigned to MSI Level 2. Metabolite quantification was performed in multiple reaction monitoring mode (MRM) of triple quadrupole MS. Partial Least Squares-Discriminant Analysis (PLS-DA) was used to analyze the difference in data. The criteria for identifying differential metabolites were a fold change ≥2 or ≤0.5, along with a variable importance in projection (VIP) ≥ 1.

### 2.3. ThT Fluorescence Assay

IAPP was dissolved in HFIP to a concentration of 500 μM and sonicated for 2 min to homogenize the sample. It was diluted by 25 mM PBS (pH 7.4) containing 50 mM NaCl and 1% HFIP to a final concentration of 5 μM. The sample was incubated at room temperature alone or with HPA at different molar ratios and 1980 μL was mixed with 20 μL ThT (2 mM, 25 mM PBS containing 50 mM NaCl, pH 7.4) before measurement.

Experiments were performed on a Perkin Elmer 6500 fluorometer (Spokane, WA, USA). The excitation and emission wavelengths were set at 450 and 486 nm, respectively. The assay solution contained 25 mM PBS (pH 7.4), 50 mM NaCl and 20 μM ThT. All experiments were repeated at least three times. The kinetic curve of the IAPP aggregation was fitted using the Boltzmann function [27]:

F = F_0_ + ∆F/(1 + exp[−(t − φ)/τ])

where F_0_ and F are fluorescence intensity at time point zero and t, respectively. Fitting parameters are ∆F, φ and τ. An initial delay time (td,) and an aggregation rate in the evolution phase (kagg) were determined by kagg = 1/τ and td = φ − 2τ.

### 2.4. MTT Cell Toxicity Assay

IAPP (1 mM in HFIP) was kept for 24 h or more for homogenization. HPA stock solution (80 mM) was prepared using 25 mM PBS containing 50 mM NaCl (pH 7.4) as the solvent and stored at −20 °C. 3-hydroxybutyrate and 3-N-Methyl-L-histidine stock solution (8 mM) were prepared using 25 mM PBS containing 50 mM NaCl (pH 7.4) as the solvent and stored at −20 °C. IAPP solution was dried with nitrogen, irradiated with UV for half an hour, and reconstituted with serum-free 1640 medium to a final concentration of 30 μM. HPA was added proportionally.

RIN-m5F cell was purchased from Jennio Biotech (Guangzhou, China). RIN-m5F cells were cultured in 1640 medium supplemented with 10% fetal bovine serum, 100 unit per mL penicillin, and 1 μg/mL streptomycin in a humidified incubator with 5% CO_2_ at 37 °C. For cytotoxicity experiments, cells were seeded at a density of 3000 cells per well in 96-well plates and cultured for about 24 h prior to the addition of the IAPP solution. After 24 h of incubation, 3-(4,5-dimethylthiazol-2-yl)-2,5-diphenyltetrazolium bromide (MTT) (5 mg/mL) was added to each well and incubated for 4 h. After removing the medium, DMSO was added to dissolve formazan. The absorbance at 490 nm was then measured using a BioTek Syergy H1 Multi-Mode Microplate Reader (Winooski, VT, USA). The average absorbance of the serum-free 1640 medium-treated cells was set as 100% cell viability. Each treatment was repeated at least six times. All results were expressed as the mean ± S.D.

### 2.5. TEM

IAPP was dissolved in HFIP (1 mM) and kept for 24 h or more for homogenization. The obtained solution was then dried by nitrogen and reconstituted with 25 mM PBS (pH 7.4) containing 50 mM NaCl to a final concentration of 100 μM. Selected metabolite candidates were added proportionally. All samples were incubated for two days at room temperature. Sample solution was blotted onto a carbon-coated 300 mesh copper grid for 10 min and then negatively stained with tungsten phosphate for 5 min. TEM images were obtained by using a HITACHI HT7700 transmission electron microscope (Tokyo, Japan).

### 2.6. CD Spectroscopy

IAPP was dissolved in 25 mM PBS (pH 7.4) containing 50 mM NaCl and 1% HFIP to a final concentration of 20 μM. All samples incubated for 17 h at room temperature. CD spectra were recorded at 25 °C under a constant flow of N_2_ by using a JASCO-810 spectropolarimeter (Tsukuba, Japan). Data were recorded from 260 to 195 nm with a 1 mm path length. Each result is given as the average of three measurements.

### 2.7. Mass Spectrometric Analysis for HPA-IAPP Interactions

An amount of 1 mM IAPP stock solution in HFIP was diluted to a final concentration of 5 μM with water (MS grade). HPA stock solution (80 mM) was prepared in 25 mM PBS, mixed with IAPP at the indicated ratios, and incubated at room temperature for 2 days. The supernatant was injected after centrifugation. A Waters Synapt G2 Si Q-TOF mass spectrometer (Milford, MA, USA) was operated to acquire data in the positive ion mode. MS parameters were set as follows: infusion mode injection; capillary voltage: 2000 V; source temperature: 100 °C; the full MS scan range: 200–3000 *m*/*z*; and scan time: 0.5 s. MS data analysis was performed by MassLynx 4.1.

### 2.8. High-Content Imaging

IAPP stock solution was dissolved in HFIP to a final concentration of 2 mM and kept for at least 24 h for homogenization. HPA stock solution was dissolved in 25 mM PBS containing 50 mM NaCl (pH 7.4) to a final concentration of 80 mM and stored at −20 °C. IAPP solution was dried with nitrogen, irradiated with UV for half an hour, and reconstituted with serum-free 1640 medium to a final concentration of 20 μM. HPA was added at a ratio of 5:1, 10:1 and 20:1 (HAP:IAPP).

Cellular immunofluorescence technology was used to detect and quantify the expression of cleaved caspase-3. The nucleic acid staining agent Hoechst 33342 was used to locate cells and observe changes in nuclear morphology in RIN-m5F cells treated with IAPP in the absence or presence of HPA using the PerkinElmer Opera Phenix TM High-Content Screening System (Spokane, WA, USA). RIN-m5F cells were seeded at a density of 5000 cells per well in poly-D-lysine-treated 96-well plates and cultured for about 24 h at 37 °C with 5% CO2. After 12 h drug incubation, 4% Paraformaldehyde fix solution was added to each well and incubated for 15 min at room temperature. After fixation, cells were washed three times with PBS, and subsequently incubated with blocking buffer (1 × PBS/5% serum/0.3% Triton X-100) for 1 h at room temperature. Microplates were then washed three times with PBS, and reacted over night at 4 °C with 50 μL/well of cleaved caspase-3 antibody (1:500, 1 × PBS/1% BSA/0.3% Triton X-100). Microplates were then washed as described above and treated for 1 h at room temperature with 50 μL/well of a donkey anti-rabbit secondary antibody Alexa Fluor Plus 555 (1:400, 1 × PBS/1% BSA/0.3% Triton X-100). Plates were again washed as described above and incubated with Hoechst 33342 for 20 min at room temperature. Microplates were then washed as described above, the final wash was aspirated, and 50 μL/well of fresh PBS was added before imaging. Each treatment was repeated three times. All results were expressed as the mean ± SEM.

### 2.9. Image Acquisition

Images were acquired on the PerkinElmer Opera Phenix TM High-Content Screening System using Harmony Software (Harmony 5.0). Images were captured in confocal mode with a 20× air objective and the channel parameters were set as follows: Hoechst, 160 ms exposure at 100% laser power; Alexa Fluor 568, 200 ms exposure at 100% laser power.

### 2.10. Statistical Analysis

For the ThT florescence assay, Origin software (version 2022) was used to fit the curve. In addition, data visualization and statistical analysis were performed using GraphPad Prism software (v8.0.2). Statistical significance was set at *p* < 0.05. For two-group comparisons, *t*-test was used to calculate the *p*-value. For comparison among three or more groups, ANOVA was used to calculate the *p*-value.

## 3. Results

### 3.1. Metabolomic Analysis of Biological Samples

**Figure 1:**
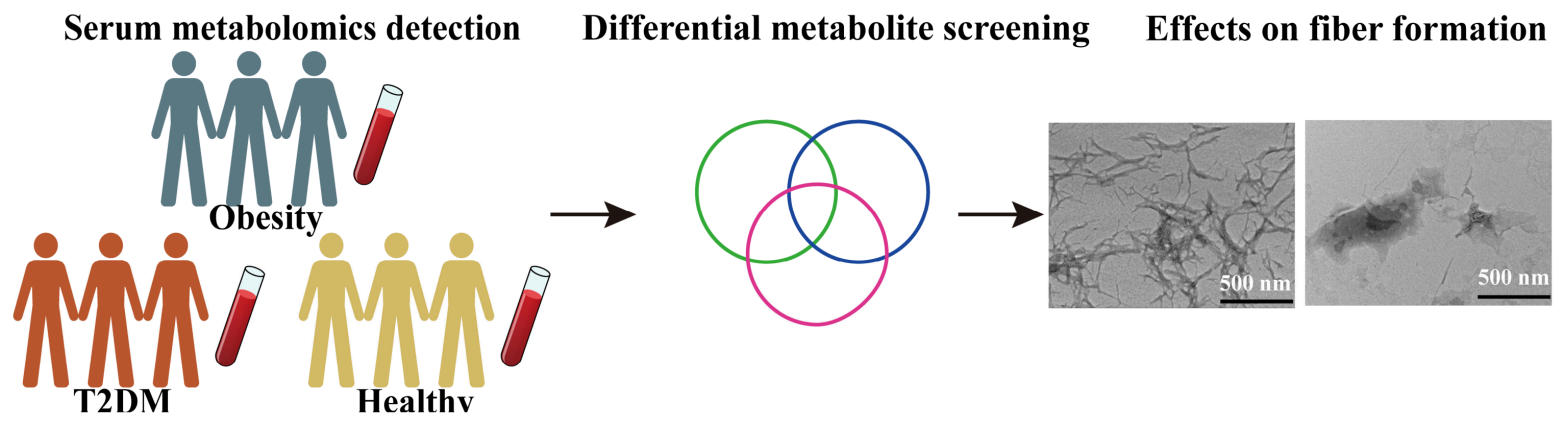
Schematic illustration of the metabolomics-guided inhibitor discovery strategy.

**Figure 2:**
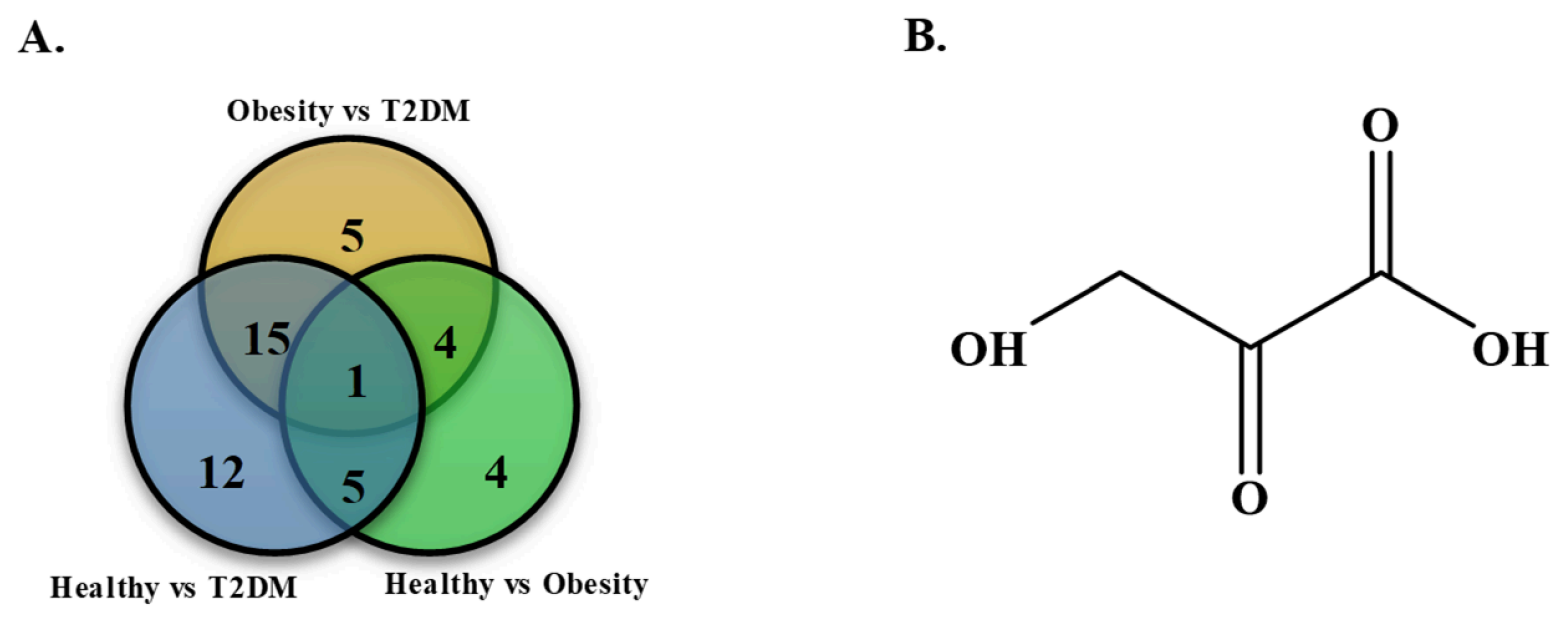
(**A**) Venn diagram of differential metabolites based on metabolomic analysis. (**B**) The molecular structure of HPA.

IAPP is a polypeptide hormone co-secreted with insulin, which has a natively unstructured form under physiological conditions. It has been recognized that under pathological conditions, IAPP undergoes misfolding to form β-sheet structures, followed by oligomerization and fibrillation in the pancreatic islets [28]. Endogenous metabolic disorders are implicated in the aggregation of IAPP, yet the exact mechanisms underlying this relationship remain elusive. In this study, we hypothesized that there could be endogenous metabolites with inhibitory effects on IAPP aggregation that remain undiscovered (Figure 1). Considering that T2DM and obesity are metabolism-related diseases, we selected serum samples from patients with T2DM and obesity, as well as healthy individuals, for differential metabolite discovery. Principal component analysis (PCA) revealed a modest trend toward group separation, although substantial overlap among the three groups was observed (Figure S1). A total of 46 differential metabolites were identified, including those previously reported to be positively associated with IAPP aggregation, such as glucose [29]. The Venn diagram in Figure 2A shows the relationships among the different groups. A total of 33 metabolites were detected in the healthy vs. T2DM group (blue circle), of which 6 overlapped with the healthy vs. obesity group (green circle), 16 overlapped with the obesity vs. T2DM group (yellow circle), and 1 metabolite was common to all three groups (details are provided in Tables S2–S4, SI). Before further investigation, six metabolites from healthy vs. obesity group (green circle) were excluded in order to eliminate the influence of obesity. Potential metabolites were further narrowed down to five T2DM-related ones according to the human metabolome database (HMDB) and literature searches, including fructose, glucose, 3-N-Methyl-L-histidine, 3-hydroxybutyrate and HPA (Table 1). Considering the well-recognized negative effects of fructose and glucose on T2DM [30,31], they were excluded. Consequently, 3-N-Methyl-L-histidine, 3-hydroxybutyrate and HPA were selected for further investigation.

### 3.2. HPA Inhibits IAPP Fibril Formation In Vitro

**Figure 3:**
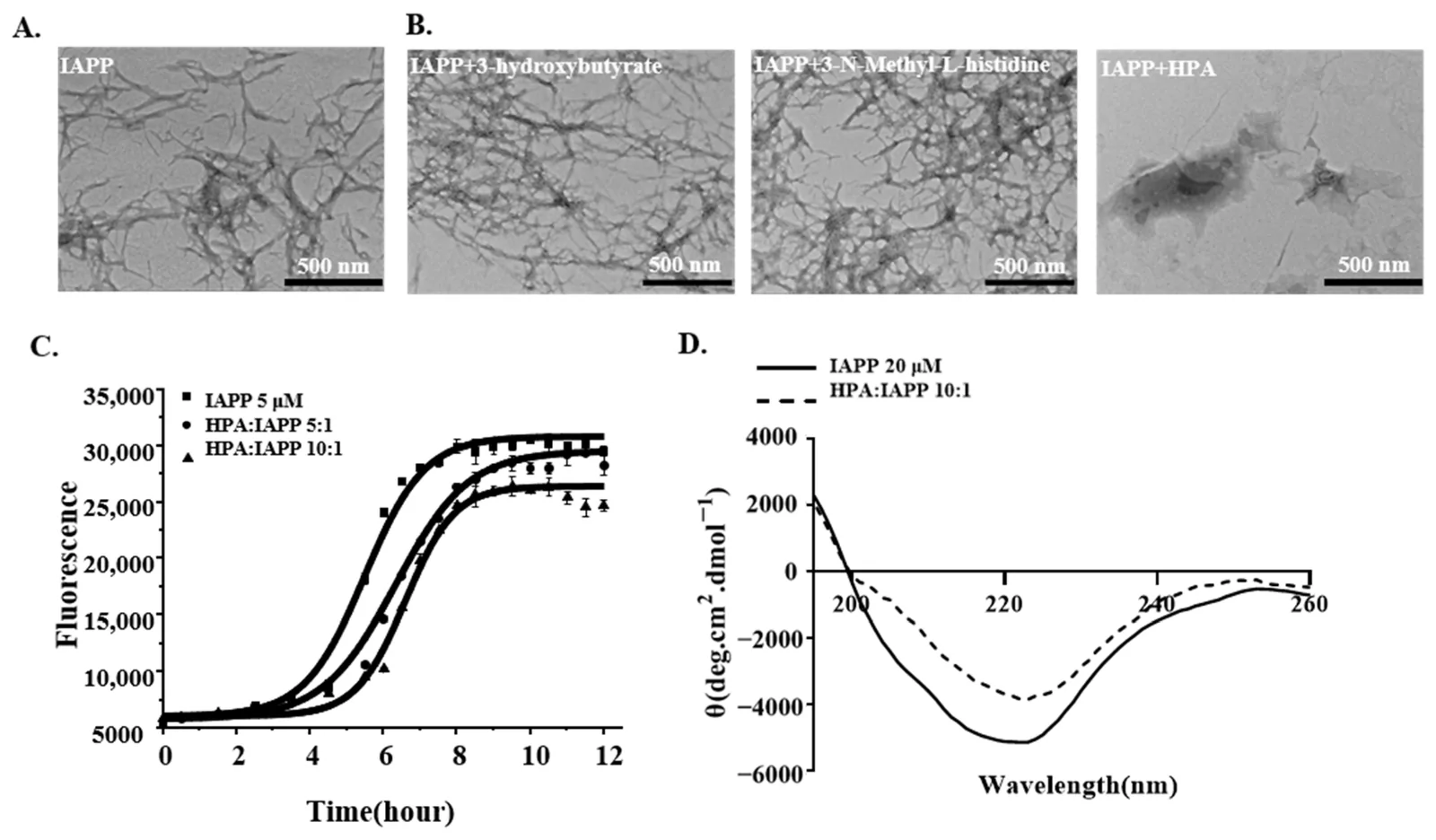
(**A**) TEM image of 100 μM IAPP alone after incubation in 25 mM PBS (pH 7.4) containing 50 mM NaCl buffer at room temperature for 2 days. (**B**) TEM image of a 10:1 molar ratio mixture of 3-hydroxybutyrate, 3-N-Methyl-L-histidine, HPA and IAPP after incubation in 25 mM PBS (pH 7.4) containing 50 mM NaCl buffer at room temperature for 2 days. Scale bars represent 500 nm. (**C**) Fluorescence thioflavin-T-binding assays of 5 μM IAPP alone (square); a 5:1 molar ratio mixture of HPA and IAPP (circle); a 10:1 molar ratio mixture of HPA and IAPP (triangle). The kinetic curves were fitted using a Boltzmann function, and the derived kinetic parameters together with their associated fitting errors are summarized in Table S5. (**D**) Far-UV CD spectrum of IAPP and HPA-IAPP mixture.

Initially, the influence of these three selected metabolites on the formation of IAPP fibrils was evaluated. The TEM image in Figure 3A shows that IAPP alone generated mature fibrils with typical morphology after incubation for 2 days at room temperature. Similar fibril formation was observed after incubation with 3-hydroxybutyrate and 3-N-Methyl-L-histidine (Figure 3B), suggesting that these two metabolites had no significant impact on preventing fibril formation. In contrast, the morphology of IAPP fibrils was markedly altered to amorphous aggregates in the presence of HPA (Figure 3B), suggesting that HPA (chemical structure shown in Figure 2B) may inhibit the formation of IAPP fibrils. The effect of HPA on the oligomerization kinetics of IAPP was subsequently monitored using a ThT fluorescence-based assay. The results show that for 5 μM IAPP alone, the fluorescence intensity over time fitted an “S”-shaped curve (Figure 3C), consistent with the typical fibril growth pattern [8]. A lag phase of approximately 3.85 h was observed for monomeric IAPP to form oligomeric intermediates. Oligomers formed rapidly, and the accumulation of β-sheet structures led to a sharp increase in fluorescence intensity, reaching a maximum value of approximately 31,000 at around 8 h, corresponding to the saturation phase. In comparison, the lag phase was prolonged to approximately 4.34 h upon incubation with HPA at a molar ratio of 5:1 (HPA:IAPP), indicating that HPA delayed the oligomerization process of IAPP aggregation. In addition, the final fluorescence intensity at the saturation phase was reduced to approximately 29,000, indicating a decreased overall β-sheet content upon the addition of HPA. Furthermore, at a molar ratio of 10:1 (HPA:IAPP), the lag phase was further extended to approximately 5.25 h, and the maximum fluorescence intensity was reduced to approximately 26,000. Collectively, the ThT assay results demonstrated that HPA inhibited IAPP aggregation by interfering with the oligomerization process in a dose-dependent manner. On this basis, far-UV circular dichroism (CD) measurements revealed a characteristic β-sheet signal around 220 nm for both IAPP alone and the IAPP–HPA mixture (Figure 3D). The reduced CD signal intensity of the IAPP–HPA mixture indicated that the addition of HPA decreased the population of β-sheet structures. Taken together, these results demonstrate that HPA effectively inhibits IAPP fibrillation, likely by suppressing oligomer formation.

### 3.3. HPA Reduces IAPP-Induced Cytotoxicity

**Figure 4:**
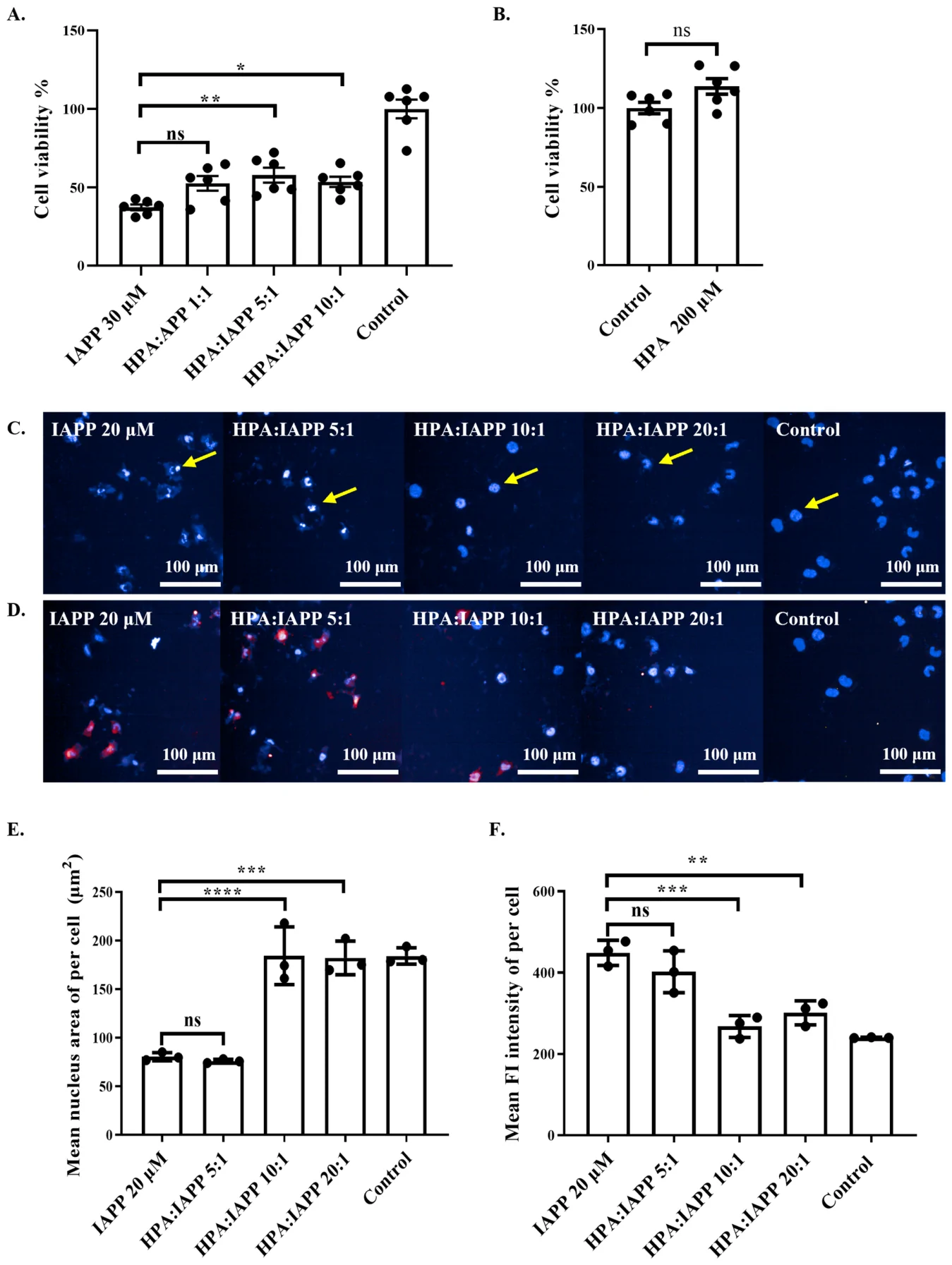
(**A**) RINm5F cell viability in the presence of IAPP and HPA mixture, as determined by MTT assay; normal cell without any treatment was used as control; ANOVA was used to calculate the *p* value. *, *p* < 0.05; **, *p* < 0.01. (**B**) HPA has no significant effect on the viability of RINm5F cells; *t*-test was used to calculate the *p*-value. (**C**) High-content images of nuclear morphology in the presence of IAPP and HPA mixture. Nuclei were stained with Hoechst 33342 (blue color); yellow arrows indicate the typical nuclear morphology for each group. (**D**) High-content images of cleaved caspase-3 cellular immunofluorescence (red) in the presence of IAPP and HPA mixture. The images were captured with a 20× air objective. Scale bars represent 100 μm. (**E**) Mean nuclear area per well of RINm5F in the presence of an IAPP and HPA mixture, as determined using a high-content imager; normal cell without any treatment was used as control; ANOVA was used to calculate the *p* value. ***, *p* < 0.001, ****, *p* < 0.0001. Each black dot represents the data from one individual well. (**F**) Cleaved caspase-3 expression level per well in the presence of an IAPP and HPA mixture, as determined using a high-content imager. ANOVA was used to calculate the *p* value. **, *p* < 0.01, ***, *p* < 0.001. All data are presented as mean ± SEM. Each black dot represents the data from one individual well.

Next, we explored whether HPA could mitigate the cytotoxicity induced by IAPP oligomers. RIN-m5F cells were selected for this study, and cell viability was assessed using an MTT assay. The results showed that a 24 h incubation with IAPP alone (30 μM) induced significant cytotoxicity, reducing cell viability to 37% (Figure 4A). Cell viability slightly increased upon the addition of HPA at a molar ratio of 1:1 (HPA:IAPP), and this increase became significantly more pronounced at a molar ratio of 5:1, with cell viability reaching 58%. Meanwhile, HPA alone had no significant effect on the viability of RIN-m5F cells (Figure 4B), further confirming that the observed increase in cell viability resulted from the inhibitory effect of HPA on IAPP aggregation. Importantly, based on previous reports indicating that oligomers are the primary contributors to cytotoxicity, the observed recovery in cell viability suggests that HPA predominantly affects the early stages of IAPP aggregation by targeting the oligomerization process, consistent with the ThT and CD results. Furthermore, no significant increase in cell viability was observed upon the addition of 3-N-methyl-L-histidine or 3-hydroxybutyrate at a molar ratio of 10:1 (Figure S2), consistent with the TEM results (Figure 3B) showing that these two metabolites had no effect on IAPP aggregation.

Endoplasmic reticulum stress-induced apoptosis mediated by caspase-3 has been reported as a major cause of cell loss in amyloid-related diseases [37,38]. We therefore proposed that a similar mechanism may underlie IAPP-induced cytotoxicity, which was further examined by immunofluorescence staining. The results showed that cells treated with 20 μM IAPP alone exhibited intense blue fluorescence and nuclear condensation (Figure 4C), indicating nuclear shrinkage and fragmentation. Meanwhile, high levels of cleaved caspase-3 expression were also detected (red fluorescence in Figure 4D, IAPP 20 μM), indicating the occurrence of apoptosis. Upon the addition of HPA, nuclear fragmentation and cleaved caspase-3 expression were significantly reduced in a dose-dependent manner. At a molar ratio of 20:1 (HPA:IAPP), nuclear morphology was largely restored to a normal state, displaying uniform blue fluorescence with oval-shaped nuclei and well-defined boundaries, comparable to the control group (Figure 4C, Control). The marked reduction in cleaved caspase-3 further confirmed the alleviation of apoptosis. Figure 4E illustrates that the average nuclear area was recovered upon the addition of HPA at the molar ratios of 10:1 (HPA:IAPP) and above, compared with the IAPP-treated group. Moreover, the averaged fluorescence intensity gradually decreased until significant difference was realized at the molar ratios of 10:1 (HPA:IAPP) and above, which confirmed that cleaved caspase-3 expression was reduced by HPA (Figure 4F). These findings are consistent with the MTT assay results, demonstrating that HPA effectively reduces IAPP-induced apoptosis.

### 3.4. HPA-IAPP Interaction Analysis Using MS

**Figure 5:**
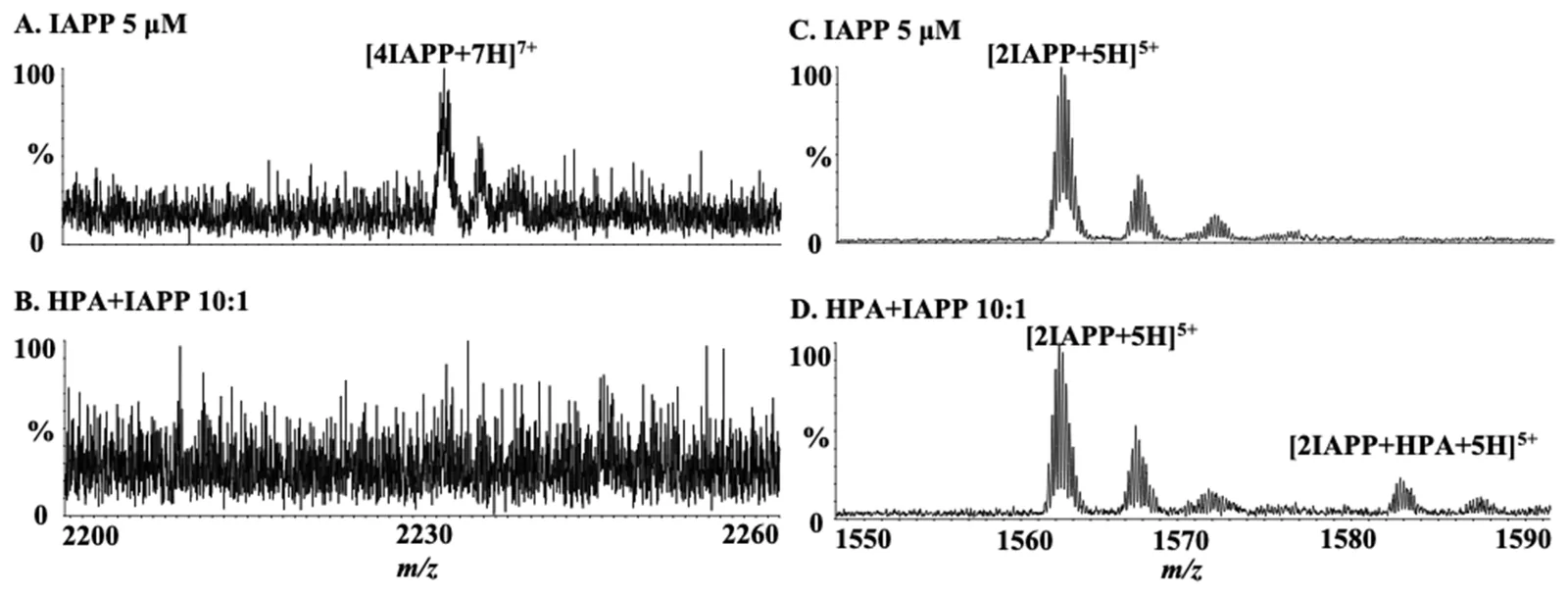
Mass spectrometric analysis of HPA–IAPP interactions. (**A**) Mass spectrum of IAPP (5 μM) showing the IAPP tetramer peak at *m*/*z* 2231.6. (**B**) Mass spectrum of IAPP incubated with HPA at a molar ratio of 1:10 (IAPP: HPA). (**C**) Mass spectrum showing the IAPP dimer peak at *m*/*z* 1562.2. (**D**) Mass spectrum showing the IAPP–HPA complex peak at *m*/*z* 1581.7.

Mass spectrometry (MS) is widely used for the characterization of protein oligomers and polymers [39,40,41]. We proposed that HPA exerts its inhibitory effect through interactions with IAPP oligomers, which was subsequently examined using MS analysis. For IAPP alone, oligomeric species with oligomerization numbers (denoted as n) ranging from 2 to 6 were readily detected (Figure S3). The addition of HPA markedly affected further oligomerization, as evidenced by a pronounced decrease in the MS signal intensity of higher-order oligomers (*n* ≥ 4). As shown by comparison between Figure 5A,B, the +7 charged ion of the IAPP tetramer at *m*/*z* 2231.6 exhibited an almost complete loss of signal intensity upon the addition of HPA. Similarly, the MS signal intensities of IAPP pentamers and hexamers were significantly reduced or became undetectable upon the addition of HPA (Figure S4). Meanwhile, direct interactions between HPA and IAPP oligomers were observed. Taking the +5 charged ion at *m*/*z* 1562.2, corresponding to the IAPP dimer, as an example (Figure 5C), the mixture with HPA yielded an additional +5 charged ion at *m*/*z* 1581.7 (Figure 5D), exhibiting a mass shift of approximately +98 Da, close to the molecular weight of a single HPA molecule. Figure S5A shows that collision-induced dissociation (CID) of the *m*/*z* 1562.2 ion predominantly yielded doubly and triply charged ions corresponding to the IAPP monomer. For CID of the *m*/*z* 1581.7 ion (Figure S5B), in addition to fragment ions corresponding to the IAPP monomer, an additional fragment ion at *m*/*z* 2001.5 was observed, corresponding to the IAPP monomer adducted with HPA and exhibiting a similar +98 Da mass shift. Notably, hydrogen bonding and electrostatic interactions are the most probable modes of interaction based on the structural characteristics of HPA and IAPP [42]. The mass variation may arise from MS signal fluctuations. The possibility of covalent binding cannot be completely excluded. Interestingly, HPA adduction was detected only for IAPP oligomers with low oligomerization numbers (*n* < 4; Figure S6), implying that HPA preferentially interacts with oligomers formed at the early stages of IAPP aggregation. Taken together with the kinetic data from ThT assays, these results indicate that HPA inhibits IAPP fibril formation by specifically interacting with low-order oligomers (*n* < 4), thereby preventing their progression to higher-order aggregates and fibrils.

## 4. Discussion

In this study, HPA was identified as a candidate metabolite capable of inhibiting IAPP aggregation. ThT fluorescence, TEM imaging, CD spectroscopy, cell viability assays, and native mass spectrometry consistently demonstrated that HPA suppresses the formation of mature amyloid fibrils by interacting with early-stage IAPP oligomers, thereby reducing IAPP-induced cytotoxicity. These findings suggest that endogenous metabolites may participate in the regulation of amyloid formation and provide a metabolomics-guided strategy for identifying modulators of protein aggregation. Despite these encouraging findings, several limitations should be considered when interpreting the results. The untargeted metabolomics analysis was conducted using a relatively small clinical cohort, which inevitably limits the statistical power and increases the possibility of false-positive discoveries. To improve the robustness of candidate selection, metabolites were not prioritized solely according to statistical significance but were further evaluated based on HMDB annotation, published literature, disease relevance, and subsequent functional validation. Nevertheless, larger independent cohorts together with targeted metabolomics analyses will be helpful to confirm the differential abundance of HPA and further establish its clinical significance.

Another important consideration is that the HPA concentrations used in the aggregation assays were selected to characterize its anti-aggregation activity and therefore do not reflect physiological circulating concentrations. Consequently, our results demonstrate that HPA inhibits IAPP aggregation under the experimental conditions employed in this study but do not establish that a comparable inhibitory effect occurs under endogenous conditions. Although HPA was found to be elevated in patients with T2DM, this increase may represent a compensatory response to metabolic stress rather than a mechanism sufficient to prevent amyloid formation. During diabetes progression, excessive synthesis and secretion of IAPP may exceed the inhibitory capacity of endogenous HPA, allowing amyloid deposition to continue despite the activation of this potential protective response. Whether endogenous HPA reaches concentrations capable of modulating IAPP aggregation in vivo, therefore, remains an open question. Likewise, although HPA stabilizes early IAPP oligomers, the biological fate of these HPA-bound complexes has not yet been determined. Whether they possess distinct structural properties, altered cytotoxicity, or different cellular clearance pathways requires further investigation before the physiological significance of HPA can be fully understood. It should also be noted that HPA is primarily recognized as an intracellular metabolic intermediate involved in serine–glycine metabolism and central carbon metabolism rather than as an extracellular signaling molecule. Therefore, the present study mainly demonstrates that HPA is capable of directly inhibiting extracellular IAPP aggregation under the experimental conditions employed. Whether endogenous HPA reaches sufficient extracellular concentrations within the pancreatic islet microenvironment, or whether intracellular HPA can modulate IAPP aggregation during peptide processing in β-cell secretory granules, remains unknown and warrants further investigation. In addition, although native MS demonstrated that HPA preferentially associates with low-order IAPP oligomers, the molecular basis of this interaction remains incompletely understood. The current data do not allow us to distinguish conclusively between transient non-covalent interactions and covalent modification, nor do they identify the binding site of HPA on IAPP. Future structural studies, including targeted molecular docking together with complementary biophysical approaches, will therefore be essential to elucidate the binding mechanism and provide a more comprehensive understanding of how HPA suppresses IAPP aggregation. Such mechanistic insights may also facilitate the rational development of endogenous metabolite-inspired therapeutics for amyloid-related diseases.

To the best of our knowledge, endogenous metabolites that directly inhibit IAPP aggregation have rarely been reported. Among endogenous molecules, insulin is the best-characterized physiological inhibitor of IAPP aggregation. In contrast, most reported anti-amyloid inhibitors are natural polyphenols, such as EGCG, curcumin, resveratrol, and myricetin, or synthetic small molecules. Although these compounds show promising in vitro activity, their therapeutic application is often limited by poor bioavailability, low stability, and uncertain physiological relevance. HPA therefore represents, to the best of our knowledge, the first endogenous metabolite identified through a metabolomics-guided strategy with direct inhibitory activity against IAPP aggregation. Although its physiological relevance and in vivo efficacy remain to be established, our findings highlight the potential of metabolomics-guided approaches for discovering endogenous modulators of amyloid aggregation.

## 5. Conclusions

In conclusion, this study establishes a metabolomics-guided strategy to identify endogenous metabolites involved in the regulation of IAPP aggregation in the context of T2DM. By integrating clinical metabolomics, biophysical characterization, cytotoxicity evaluation, and mass spectrometry analysis, we identified HPA as a candidate endogenous metabolite that modulates the early stages of IAPP aggregation. Our findings demonstrate that HPA attenuates IAPP fibril formation by interacting with early oligomeric species and reducing their progression toward mature amyloid structures, thereby alleviating IAPP-associated cytotoxicity under experimental conditions.

Although the physiological relevance and precise molecular mechanism of HPA–IAPP interaction require further validation, this study provides evidence that metabolic alterations in T2DM may influence amyloid formation through endogenous regulatory mechanisms. These findings highlight the importance of exploring metabolite–protein interactions in amyloid diseases and provide a foundation for future studies investigating the role of HPA and other endogenous metabolites in the regulation of IAPP pathogenesis.

## Figures and Tables

**Table 1:** Detailed information of T2DM-related metabolites.

Compounds	CAS Number	Up/Down Regulation	References
D-Fructose	53188-23-1	up	[32]
D-Glucose	50-99-7	up	[33,34]
3-Hydroxybutyrate	625-72-9	up	[35]
β-Hydroxypyruvic Acid	1113-60-6	up	[25]
3-Methylhistidine	368-16-1	up	[36]

## Data Availability

Data available on request due to restrictions.

## Supplementary Materials

The following supporting information can be downloaded at: https://www.mdpi.com/article/10.3390/metabo16080549/s1, Figure S1. PCA plot of metabolomic profiles from healthy controls, obesity, and T2DM groups. Each point represents an individual sample, and the shaded ellipses indicate the 95% confidence interval for each group. Figure S2. RINm5F cells viability in the presence of IAPP and 3-N-Methyl-L-histidine/3-hydroxybutyrate mixture and measured by MTT assay. Normal cell was set as control. Figure S3. Mass spectrum of IAPP oligomers. Figure S4. Mass spectra of IAPP tetramer at *m*/*z* 1736.1 with (**A**) IAPP at 5 μM; (**B**) HPA-IAPP mixture at a molar ratio of 10:1; IAPP tetramer at *m*/*z* 1420.3 with (**C**) IAPP at 5 μM; (**D**) HPA-IAPP mixture at a molar ratio of 10:1; IAPP pentamer at *m*/*z* 1775.3 with (**E**) IAPP at 5 μM; (**F**) HPA-IAPP mixture at a molar ratio of 10:1; and IAPP hexamer at *m*/*z* 2343.1 with (**G**) IAPP at 5 μM; (**H**) HPA-IAPP mixture at a molar ratio of 10:1. Figure S5. CID spectra of (**A**) *m*/*z* 1562.2 and (**B**) *m*/*z* 1581.7. Figure S6. Mass spectra of IAPP at 5 μM (**A**) IAPP trimer at *m*/*z* 1464.7; and (**C**) IAPP trimer at *m*/*z* 1673.8. Mass spectra of HPA-IAPP complex at molar ratio of 10:1 (**B**) IAPP-HPA complex at *m*/*z* 1476.9; and (**D**) IAPP-HPA complex at *m*/*z* 1687.7. Table S1. Demographic detail of the subjects recruited for the metabolomics study. Table S2. Differential metabolites in Healthy vs T2DM. Table S3. Differential metabolites in Healthy vs Obesity. Table S4. Differential metabolites in Obesity vs T2DM. Table S5. Boltzmann fitting parameters and associated fitting errors for the ThT fluorescence kinetic curves shown in Figure 3C.

## Abbreviations

The following abbreviations are used in this manuscript:

T2DM	T2DM Type 2 diabetes mellitus
TEM	Transmission electron microscopy
HMDB	Human metabolome database
MSI	Metabolomics Standards Initiative
ThT	Thioflavin T
HPA	β-hydroxypyruvic acid
EGCG	Epigallocatechin-3-gallate
MS	Mass spectrometry
