# Supplementary material for: A Metabolomics-Based Strategy for Identifying Endogenous Inhibitors of IAPP Aggregation
Source: Metabolites. 2026 Aug 3;16(8):549. doi: 10.3390/metabo16080549 (PMC13515482; doi:10.3390/metabo16080549)
Supplement: Supplementary file 1 [file metabolites-16-00549-s001.zip › metabolites-4382249-supplementary.pdf]

Article

A Metabolomics-Based Strategy for Identifying Endogenous Inhibitors of IAPP Aggregation

Dandan Xia [1,2,3,†], Liubao Gu [4,†], Lei Yang [3], Jiaojiao Hu [3], Xiaowei Xu [5], Dechen Jiang [2], Qiuling Zheng [3,*] and Bing Wan [1,*]

1 Department of Respiratory and Critical Care Medicine, The Affiliated Jiangning Hospital of Nanjing Medical University, Nanjing 211100, China; dg21240118@smail.nju.edu.cn
2 The State Key Lab of Analytical Chemistry for Life Science, Chemistry and Biomedicine Innovation Center (ChemBIC), School of Chemistry and Chemical Engineering, Nanjing University, Nanjing 210093, China
3 Department of Pharmaceutical Analysis, School of Pharmacy, China Pharmaceutical University, Tongjiaxiang #24, Nanjing 210009, China
4 Division of Clinical Epidemiology, Geriatric Hospital of Nanjing Medical University, Nanjing 210009, China; liubao_gu@njmu.edu.cn
5 Institute of Innovative Drug Discovery and Development, China Pharmaceutical University, Tongjiaxiang #24, Nanjing 210009, China
* Correspondence: qiuling_zheng@cpu.edu.cn (Q.Z.); bingwan76@njmu.edu.cn (B.W.)
† These authors contributed equally to this work.

Academic Editor: Stewart Graham

Received: 1 June 2026
Revised: 10 July 2026
Accepted: 28 July 2026
Published: 3 August 2026

Copyright: © 2026 by the authors. Submitted for possible open access publication under the terms and conditions of the Creative Commons Attribution (CC BY) license.

Additional Figures and Tables

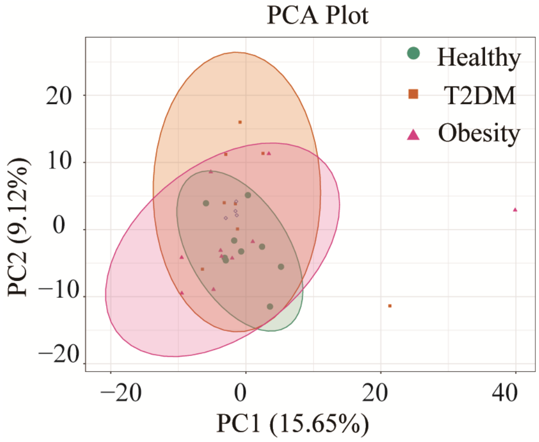

Figure S1. PCA plot of metabolomic profiles from healthy controls, obesity, and T2DM groups. Each point represents an individual sample, and the shaded ellipses indicate the 95% confidence interval for each group.

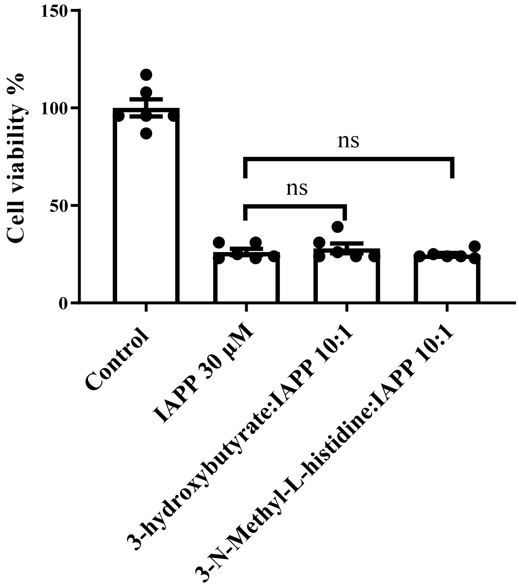

Figure S2. RINm5F cells viability in the presence of IAPP and 3-N-Methyl-L-histidine/3-hydroxybutyrate mixture and measured by MTT assay. Normal cell was set as control.

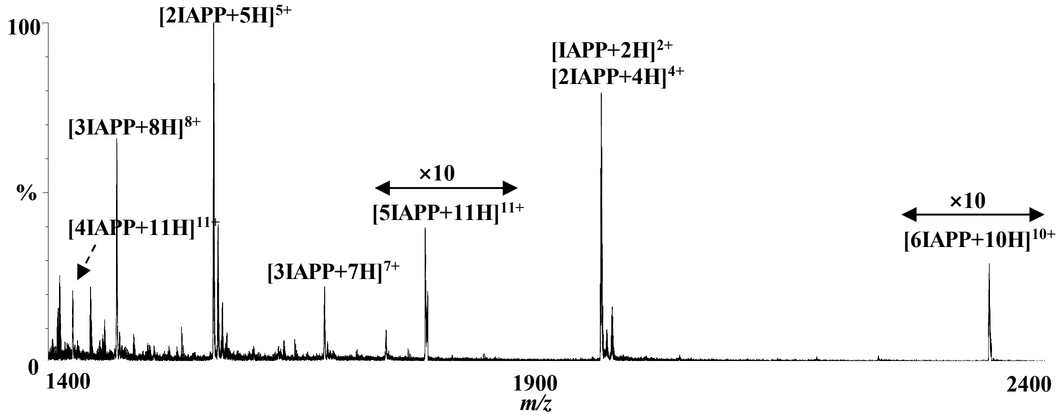

Figure S3. Mass spectrum of IAPP oligomers.

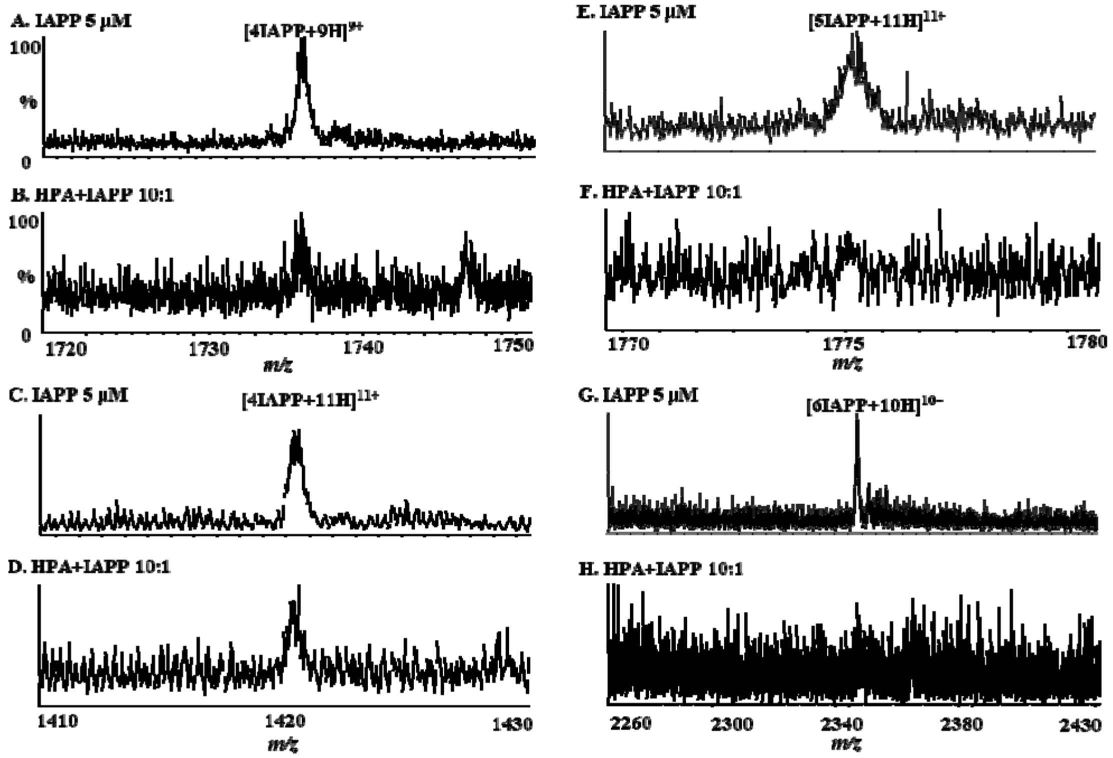

Figure S4. Mass spectra of IAPP tetramer at *m*/*z* 1736.1 with (A) IAPP at 5 μM; (B) HPA-IAPP mixture at a molar ratio of 10:1; IAPP tetramer at *m*/*z* 1420.3 with (C) IAPP at 5 μM; (D) HPA-IAPP mixture at a molar ratio of 10:1; IAPP pentamer at *m*/*z* 1775.3 with (E) IAPP at 5 μM; (F) HPA-IAPP mixture at a molar ratio of 10:1; and IAPP hexamer at *m*/*z* 2343.1 with (G) IAPP at 5 μM; (H) HPA-IAPP mixture at a molar ratio of 10:1.

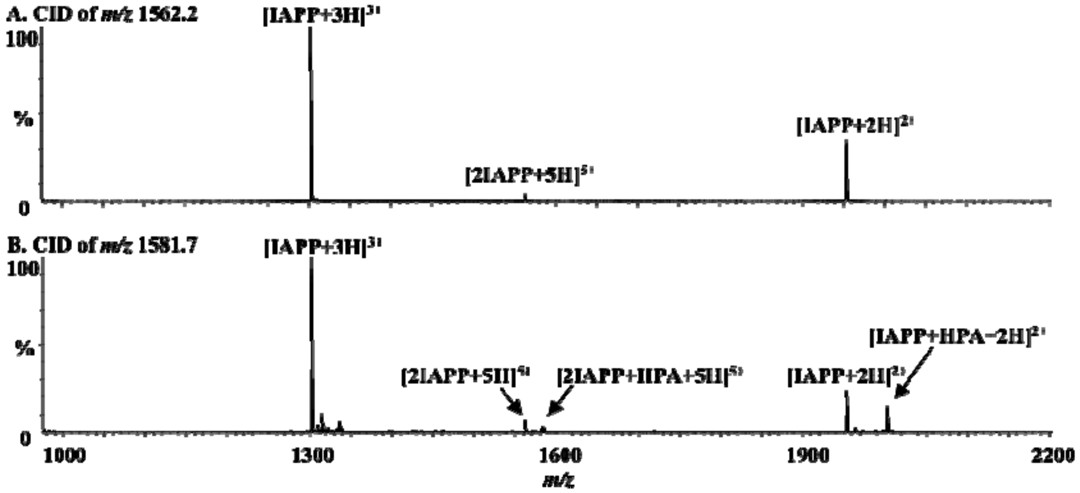

Figure S5. CID spectra of (A) *m/z* 1562.2 and (B) *m/z* 1581.7.

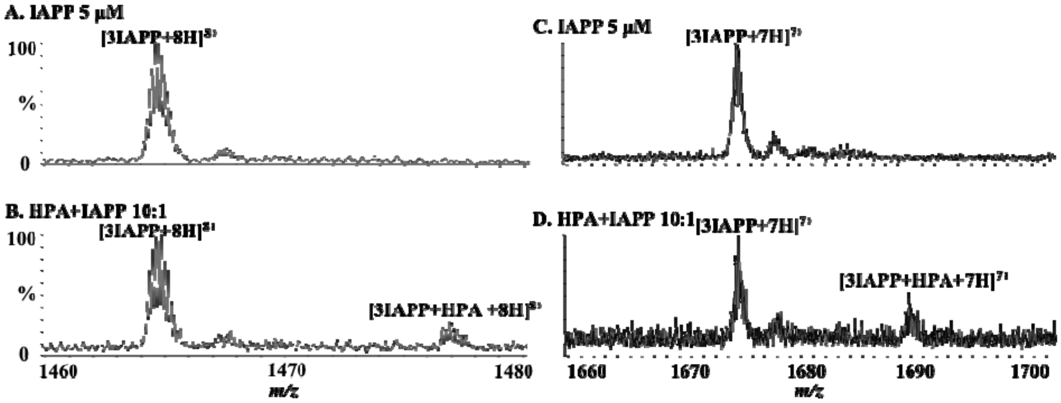

Figure S6. Mass spectra of IAPP at 5 μM (A) IAPP trimer at *m/z* 1464.7; and (C) IAPP trimer at *m/z* 1673.8. Mass spectra of HPA-IAPP complex at molar ratio of 10:1 (B) IAPP-HPA complex at *m/z* 1476.9; and (D) IAPP-HPA complex at *m/z* 1687.7.

Table S1. Demographic detail of the subjects recruited for the metabolomics study.

Cohort	Healthy	T2DM	Obesity
Sex (Male/Female)	7/2	4/4	9/1
Age (years, Mean ± SD)	71.8±7.7	74.75±5.1	76.4±8.9
Range of Age (years)	66-86	67-85	66-89
BMI (kg/m², Mean ± SD)	19.92±2.866	21.33±2.276	30.50±2.331
Range of BMI (kg/m²)	13.80-22.00	17.60-23.70	28.40-35.70

Table S2. Differential metabolites in Healthy vs T2DM

Number	Compound	FC	P value
1	Theobromine	0.23	0.09
2	Taurocholic Acid	3.48	0.07
3	Hyodeoxycholic Acid	0.33	0.21
4	Glycocholic Acid	2.36	0.17
5	1-Methylxanthine	0.28	0.07
6	2-Hydroxy-6-Aminopurine	26.83	0.26
7	D-Fructose	2.24	0.16
8	D-Glucose	2.14	0.16
9	1,3-Dimethyluric Acid	0.29	0.07
10	3-Hydroxybutyrate	2.11	0.07
11	Ethyl 3,4-Dihydroxybenzoate	32.76	0.14
12	Malonicacid	2.10	0.07
13	Thromboxane B2	0.22	0.01
14	12-Hete	0.49	0.14
15	EPA [5Z,8Z,11Z,14Z,17Z-eicosapentaenoic acid]	2.55	0.16
16	3-(3-Hydroxyphenyl) Propionate Acid	2.23	0.08
17	Methyl 3-Hydroxyphenylacetate	2.07	0.11
18	D-Glucopyranose	2.39	0.09
19	β-Hydroxypyruvic Acid	2.12	0.07
20	INDOLE-3-CARBINOL	0.39	0.02
21	(±)12-HETE [(±)12-hydroxy-5Z,8Z,10E,14Z-eicosatetraenoic acid]	0.37	0.07
22	11(S)-HETE [11S-hydroxy-5Z,8Z,12E,14Z-eicosatetraenoic acid]	0.34	0.05
23	11,12-EET [(±)11,(12)-epoxy-5Z,8Z,14Z-eicosatrienoic acid]	0.30	0.01
24	3-N-Methyl-L-Histidine	2.33	0.10
25	N-Propionylglycine	0.40	0.0039
26	1,7-Dimethylxanthine	0.26	0.03
27	3-Methylxanthine	0.43	0.19
28	7-Methylxanthine	0.44	0.09
29	Indole-3-Carboxaldehyde	0.37	0.02
30	Trans-3-Hydroxycotinine	0.48	0.25
31	Uridine 5-Monophosphate	2.30	0.01
32	Isoquinoline	0.37	0.03

33	Pterine	3.62	0.01

Table S3. Differential metabolites in Healthy vs Obesity

Number	Compound	FC	P value
1	P–Hydroxyphenyl Acetic Acid	2.13	0.03
2	Taurocholic Acid	3.70	0.04
3	TXB2 [9α,11,15S-trihydroxythromba-5Z,13E-dien-1-oic acid]	0.40	0.16
4	12-Hete	0.44	0.06
5	Methylstearate	0.47	2.68E^{-9}
6	(±)12-HETE [(±)12-hydroxy-5Z,8Z,10E,14Z-eicosatetraenoic acid]	0.40	0.13
7	(±)4-HDHA [(±)4-hydroxy-5E,7Z,10Z,13Z,16Z,19Z-docosahexaenoic acid]	0.50	0.19
8	11(S)-HETE [11S-hydroxy-5Z,8Z,12E,14Z-eicosatetraenoic acid]	0.44	0.14
9	12,13-EpOME [(±)12(13) epoxy-9Z-octadecenoic acid]	0.14	0.0014
10	Trans-3-Hydroxycotinine	5.4E^{-6}	0.0042
11	N-METHYL (-) EPHEDRINE	0.49	0.22
12	2-Furoylglycine	0.26	0.10
13	Trimethoprim	2.42	0.07
14	1,3-Di-o-tolylguanidine	0.50	0.28

Table S4. Differential metabolites in Obesity vs T2DM

Number	Compound	FC	P value
1	Theobromine	0.16	0.07
2	Glycocholic Acid	2.29	0.13
3	1-Methylxanthine	0.16	0.05
4	2-Hydroxy-6-Aminopurine	27.06	0.14
5	L-Fucose	2.29	0.02
6	L-Rhamnose	2.35	0.02
7	1,3-Dimethyluric Acid	0.17	0.02
8	3-Hydroxybutyrate	2.79	0.0091
9	Caffeic Acid	2.01	0.43
10	Malonic acid	2.78	0.01
11	EPA [5Z,8Z,11Z,14Z,17Z-eicosapentaenoic acid]	2.42	0.17
12	β-Hydroxypyruvic Acid	2.77	0.01
13	MARMESIN	3.00	0.04
14	INDOLE-3-CARBINOL	0.37	0.03
15	12,13-EpOME [(±)12(13) epoxy-9Z-octadecenoic acid]	15.45	0.0024
16	3-N-Methyl-L-Histidine	2.22	0.11
17	1,7-Dimethylxanthine	0.20	0.07
18	7-Methylxanthine	0.32	0.02
19	Indole-3-Carboxaldehyde	0.39	0.04
20	Trans-3-Hydroxycotinine	89756	0.11
21	Isoquinoline	0.41	0.05
22	N-METHYL (-) EPHEDRINE	2.40	0.08
23	3-(2-Hydroxyphenyl) propionic acid	2.99	0.03
24	Trimethoprim	0.41	0.08
25	1,3-Di-o-tolylguanidine	2.02	0.06

Table S5. Boltzmann fitting parameters and associated fitting errors for the ThT fluorescence kinetic curves shown in Figure 3C.

Model	Boltzmann		
Equation	y = A2 + (A1-A2)/(1 + exp((x-x0)/dx))		
Plot	Mean-IAPP	Mean-HPA: IAPP 5:1	Mean-HPA: IAPP 10:1
A1	6123.96 ± 234.54	5805.87 ± 103.44	6129.68 ± 112.45
A2	30804.09 ± 978.48	29599.85 ± 596.09	26488.78 ± 877.37
x0	5.48 ± 0.16	6.33 ± 0.07	6.60 ± 0.11
dx	0.82 ± 0.09	0.99 ± 0.06	0.67 ± 0.05
Reduced Chi-Sqr	50.39	20.36	23.25
R-Square (COD)	0.98	0.99	0.99
Adj. R-Square	0.98	0.99	0.99
